# Direct Location of Organic Molecules in Framework Materials by Three-Dimensional Electron Diffraction

**DOI:** 10.1021/jacs.2c05122

**Published:** 2022-08-11

**Authors:** Meng Ge, Taimin Yang, Hongyi Xu, Xiaodong Zou, Zhehao Huang

**Affiliations:** Department of Materials and Environmental Chemistry, Stockholm University, Stockholm SE-106 91, Sweden

## Abstract

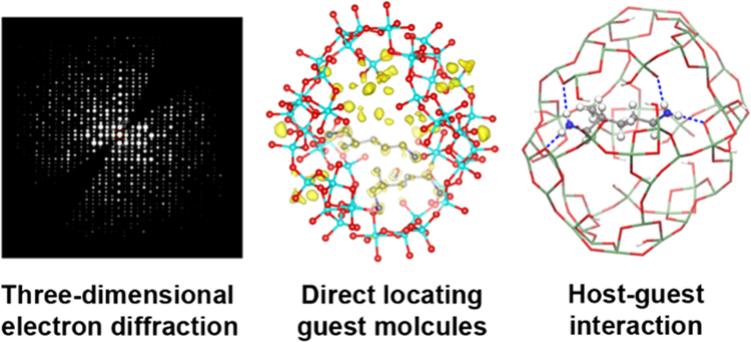

In the study of framework materials, probing interactions between frameworks and organic molecules is one of the most important tasks, which offers us a fundamental understanding of host–guest interactions in gas sorption, separation, catalysis, and framework structure formation. Single-crystal X-ray diffraction (SCXRD) is a conventional method to locate organic species and study such interactions. However, SCXRD demands large crystals whose quality is often vulnerable to, e.g., cracking on the crystals by introducing organic molecules, and this is a major challenge to use SCXRD for structural analysis. With the development of three-dimensional electron diffraction (3D ED), single-crystal structural analysis can be performed on very tiny crystals with sizes on the nanometer scale. Here, we analyze two framework materials, SU-8 and SU-68, with organic molecules inside their inorganic crystal structures. By applying 3D ED, with fast data collection and an ultralow electron dose (0.8–2.6 e^–^ Å^–2^), we demonstrate for the first time that each nonhydrogen atom from the organic molecules can be ab initio located from structure solution, and they are shown as distinct and well-separated peaks in the difference electrostatic potential maps showing high accuracy and reliability. As a result, two different spatial configurations are identified for the same guest molecule in SU-8. We find that the organic molecules interact with the framework through strong hydrogen bonding, which is the key to immobilizing them at well-defined positions. In addition, we demonstrate that host–guest systems can be studied at room temperature. Providing high accuracy and reliability, we believe that 3D ED can be used as a powerful tool to study host–guest interactions, especially for nanocrystals.

## Introduction

The family of framework materials, which have extended infinitive three-dimensional (3D) crystalline structures,^1^ has been expanding from traditional zeolites (SiO_2_) and aluminophosphates (AlPO_4_)^2,3^ to other main-block elements such as germanium, gallium, and indium-based materials^4,5^ and to the recently developed metal–organic frameworks (MOFs)^6,7^ and covalent–organic frameworks (COFs).^8^ Due to their permanent porosity and versatile properties, framework materials have attracted considerable interest and shown large potential in a wide range of applications such as gas sorption, separation, catalysis, etc.^9−15^ In the center of these materials and their associated applications, the underlying host–guest interactions play an indispensable role in understanding the performance and properties of the materials.^16−20^ Thus, to access the rich knowledge provided by host–guest interactions and guest molecules, one important task is to precisely determine their locations and molecular configurations.

In this context, single-crystal analysis provides the most accurate position, configuration, and bonding information, which are crucial for studying guest molecules such as gas molecules and organic molecules in framework materials and understanding host–guest interactions. Single-crystal X-ray diffraction (SCXRD) has been applied for such analysis in framework materials to obtain insights into gas adsorption,^21−23^ framework formation,^24^ and organic molecule structures.^25,26^ However, since SCXRD requires large and high-quality crystals, the analysis of guest molecules and host–guest interactions is often hampered by the difficulties in growing such crystals. In addition, compared to nanoscale crystals, large crystals are much easier to be damaged by, i.e., fatigue crack introduced by the interactions. While powder X-ray diffraction (PXRD) could analyze nanocrystals and has been used to provide crucial information about organic molecules in framework materials,^27,28^ their locations are usually determined by finding the best fit of the whole molecules to large blocks of density. Therefore, the accuracy is often sensitive to the challenges in PXRD analysis, such as peak overlapping and the presence of impurity. Three-dimensional electron diffraction (3D ED) has been developed as a unique technique for single-crystal analysis of tiny crystals.^29−33^ Due to the strong interaction between electrons and matter, the crystal size required for single-crystal analysis has been dramatically reduced to a few tens of nanometers, and various structures of nanosized framework materials have been determined using 3D ED.^34−37^ Because only a small piece of crystal is needed, it, therefore, opens new opportunities to tackle the aforementioned drawback of crystal growth while maintaining the accuracy of single-crystal analysis. Furthermore, small crystals are less vulnerable to cracking, and they can facilitate diffusion, which increases the occupancy of organic molecules in the pores for detection. 3DED data can provide structural information about organic molecules in framework materials and biological compounds.^38−41^ However, in the previous studies, it was challenging to reach similar reliability as SCXRD to directly locate organic molecules in the previous studies. The organic molecules are mostly identified during the refinement. It is common that not all atoms from the organic molecules can be directly located, and there could also be missing peaks in electrostatic potential maps. Each of these could hinder an accurate identification, especially for those molecules that can adopt different configurations.

With recent development in continuous 3D ED protocols,^42−46^ here, we use an ultralow electron dose (0.8–2.6 e^–^ Å^–2^) to prevent organic molecules from being damaged by the high-energy electron beam. We report ab initio location of organic molecules with the structure determination of two framework materials, SU-8 and SU-68, by continuous rotation electron diffraction (cRED). While the framework structure and organic species of SU-8 have been studied by SCXRD,^47^ those of SU-68 were solved for the first time by cRED. Being benefitted from the ultralow dose, all of the nonhydrogen atoms from both the organic molecules and the frameworks are directly obtained from the structure solution by direct methods. Different configurations of organic molecules can directly be observed after structure solution. Remarkably, atoms of the organic molecules are recognized as distinct and well-separated peaks in the difference electrostatic potential maps, showing high accuracy and reliability as we compare the results to those obtained by SCXRD. In addition, we demonstrate that organic molecules in SU-8 can be located at room temperature by cRED, which opens possibilities to investigate organic molecules at a closer state to their pristine form without considering possible structural changes introduced by low temperature.^48^ By locating organic molecules and knowing their configurations, it enables us to further investigate their interactions with the frameworks, including hydrogen bonding and van der Waals (vdW) interaction. As framework materials may result in different crystallinities, we investigate the influence of cRED data resolution cutoffs on the direct locating of organic molecules.

## Experimental Section

### Syntheses of SU-8 and SU-68

The 3D open-framework germanate SU-8 was synthesized as previously reported.^47^ In a typical hydrothermal synthesis of SU-68, 100 mg of germanium dioxide, 0.5 mL of tris(2-aminoethyl)-amine (TAEA), 0.3 mL of water, 2.0 mL of dimethylformamide (DMF), and 0.15 mL hydrofluoric acid were mixed to form a clear solution. The solution was then transferred into a Teflon-lined stainless-steel autoclave and heated at 160 °C for 7 days. The crystals were collected by filtration, washed with deionized water, and dried at room temperature. To grow single crystals of SU-68, 100 mg of germanium dioxide, 1.0 mL of TAEA, 1.0 mL of DMF, and 0.15 mL of hydrofluoric acid were used. The clear mixed solution was transferred into a Teflon-lined autoclave and heated at 160 °C for 7 days.

### cRED Data Collection

The sample was crushed in a mortar and dispersed in absolute ethanol. A droplet was then transferred onto a copper grid covered with lacey carbon and dried in air. Data were collected on a JEOL JEM 2100 microscope operated at 200 kV (Cs 1.0 mm, point resolution 0.23 nm). TEM images were recorded with a Gatan Orius 833 CCD camera (resolution 2048 × 2048 pixels, pixel size 7.4 μm). cRED data were acquired using software Instamatic,^42^ and the electron diffraction (ED) frames were recorded using a Timepix hybrid detector QTPX-262k (512 × 512 pixels, pixel size 55 μm, Amsterdam Sci. Ins.). A single-tilt holder (tilting range: −70 to +70°) was used for the data collection of SU-8 under room temperature. For SU-68, the sample was cooled to 96 K using a Gatan cryotransfer tomography holder. The area used for cRED data collection was about 1.0 μm in diameter, as defined by the selected area aperture.

### Thermogravimetric Analysis (TGA)

Crystals of SU-68 were heated on a TA Instruments Discovery TGA 5500 thermogravimetric analyzer from room temperature to 800 °C at a rate of 10 °C min^–1^ under airflow.

## Results and Discussion

**Figure 1:**
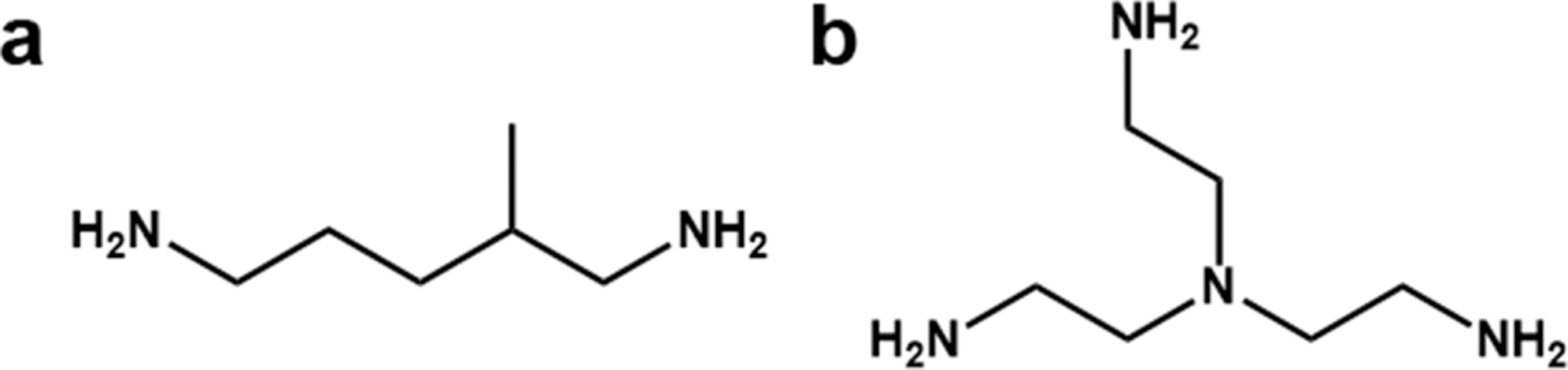
Structural formulae of (a) MPMD and (b) TAEA that are immobilized in SU-8 and SU-68, respectively.

We applied cRED on SU-8 and SU-68 crystals to locate guest molecules in the open-framework materials. The guest molecules of 2-methyl-1,5-pentanediamine (MPMD) and tris(2-aminoethyl)-amine (TAEA) are immobilized in SU-8 and SU-68 during the synthesis (Figure 1). We selected SU-8 and SU-68 for the study because they represent two typical types of framework materials. SU-8 has a 3D framework structure with the organic molecules in the pores, while the structure of SU-68 is built from 2D layers with the organic molecules located between the layers. In addition, SU-8 is block crystals and SU-68 is plate-like. Crystals with 2D morphologies usually have preferred orientations in a TEM grid, and their data completeness could be limited.

**Figure 2:**
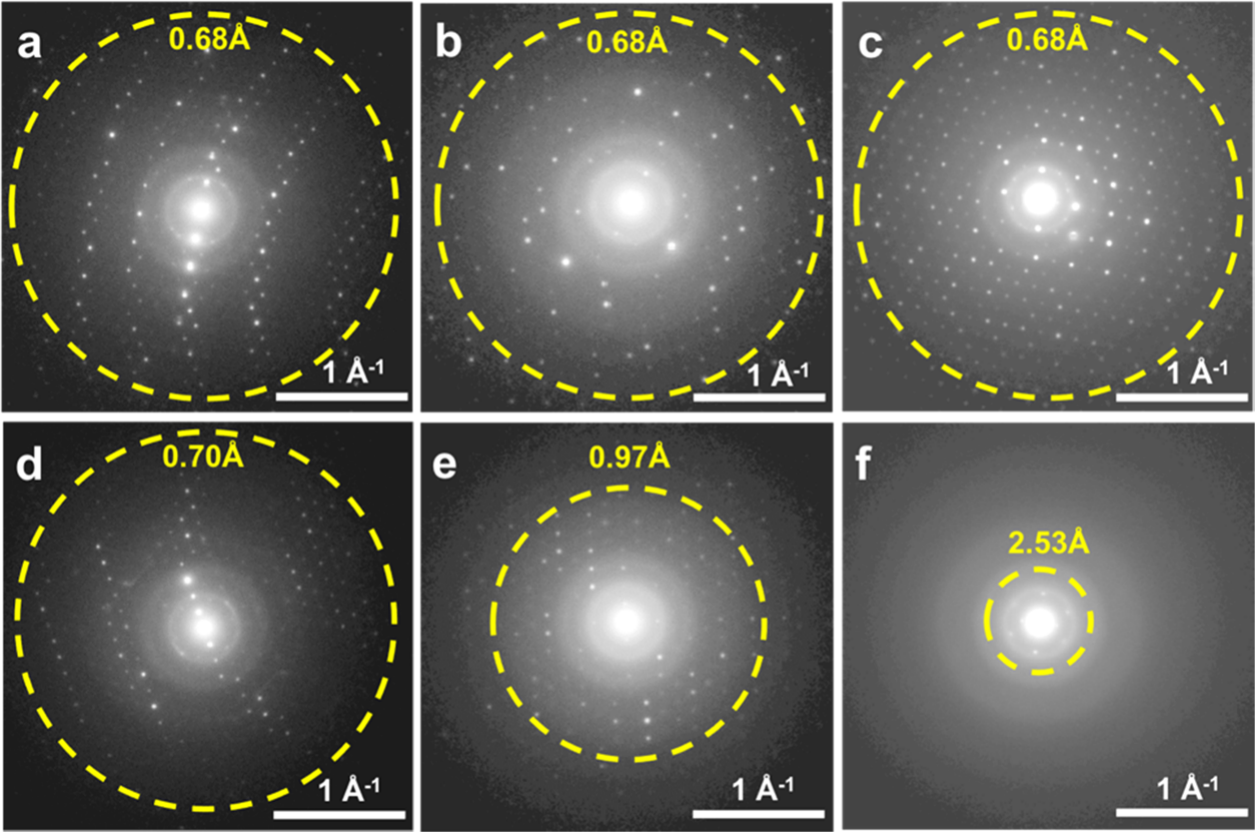
Influence of the electron dose on the data resolution of SU-68. (a–c) Starting resolutions compared to those after being exposed at an electron dose of (d) 0.6 e^–^ Å^–2^, (e) 1.8 e^–^ Å^–2^, and (f) 3.0 e^–^ Å^–2^. All of the crystals have been exposed for the same time period with different dose rates of (d) ∼0.01 e^–^ s^–1^ Å^–2^, (e) ∼0.03 e^–^ s^–1^ Å^–2^, and (f) ∼0.05 e^–^ s^–1^ Å^–2^. Different SU-68 crystals can have slightly different initial resolutions.

To obtain high-quality data for the study of organic molecules in SU-8 and SU-68, it is crucial to minimize electron beam damage. We used an ultralow electron dose rate (∼0.01 e^–^ s^–1^ Å^–2^), which was controlled by adjusting the spot size and excitation of the C2 lens. As resolution is one of the most important indicators of data quality, the advantages of using ultralow dose measurement can be observed by preventing the loss of Bragg reflections. For example, SU-68 has an initial resolution higher than 0.68 Å (Figure 2a–c). After being exposed to an electron beam at a dose of 0.6 e^–^ Å^–2^, the resolution was maintained at 0.70 Å (Figure 2d). However, using a higher dose rate of ∼0.03 and ∼0.05 e^–^ s^–1^ Å^–2^, the resolution decreased to 0.97 and 2.53 Å, respectively (Figure 2e,f), which are less favorable for studying guest molecules. In addition, to use an ultralow electron dose rate, we shorten the total time of data acquisition by applying a high rotation speed of the goniometer (0.45° s^–1^) and collecting data at a tilting range as small as 35°. Thus, in the handling of low-dose data, it is important to merge several data sets to achieve a high completeness (see the Supporting Information for more details).

### Guest Molecules in Three-dimensional Framework SU-8

**Figure 3:**
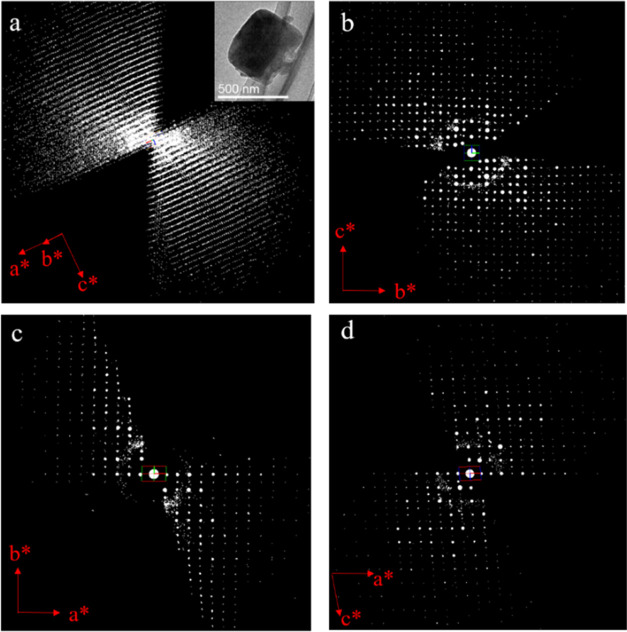
(a) Reconstructed 3D reciprocal lattice of SU-8. The inset shows the crystal morphology of SU-8 (ca. 500 nm in size). 2D slice cuts from the reconstructed 3D reciprocal lattice of SU-8 showing the (b) 0*kl*, (c) *hk*0, and (d) *h*0*l* planes.

The cRED data of SU-8 were collected on small single crystals (<1 μm) with a block morphology at room temperature (Figure 3a). High-quality cRED data were obtained with a resolution of 0.74 Å under an average dose of 2.4 e^–^ Å^–2^. From the 3D reconstructed reciprocal lattice (Figures 3 and S1), SU-8 crystallizes in a monoclinic space group **P**21̅**c** (no. 14) with unit cell parameters of *a* = 12.169(2) Å, *b* = 19.442(4) Å, *c* = 19.289(4) Å, and β = 92.45(3)° (see the Supporting Information for more details). Ab initio structure determination was applied to the cRED data sets using direct methods implemented in the SHELX software package.^49^ The positions of all nonhydrogen atoms, including guest molecules, were found directly from the structure solution. The crystallographic details of SU-8 from using cRED data are summarized in Table S1.

**Figure 4:**
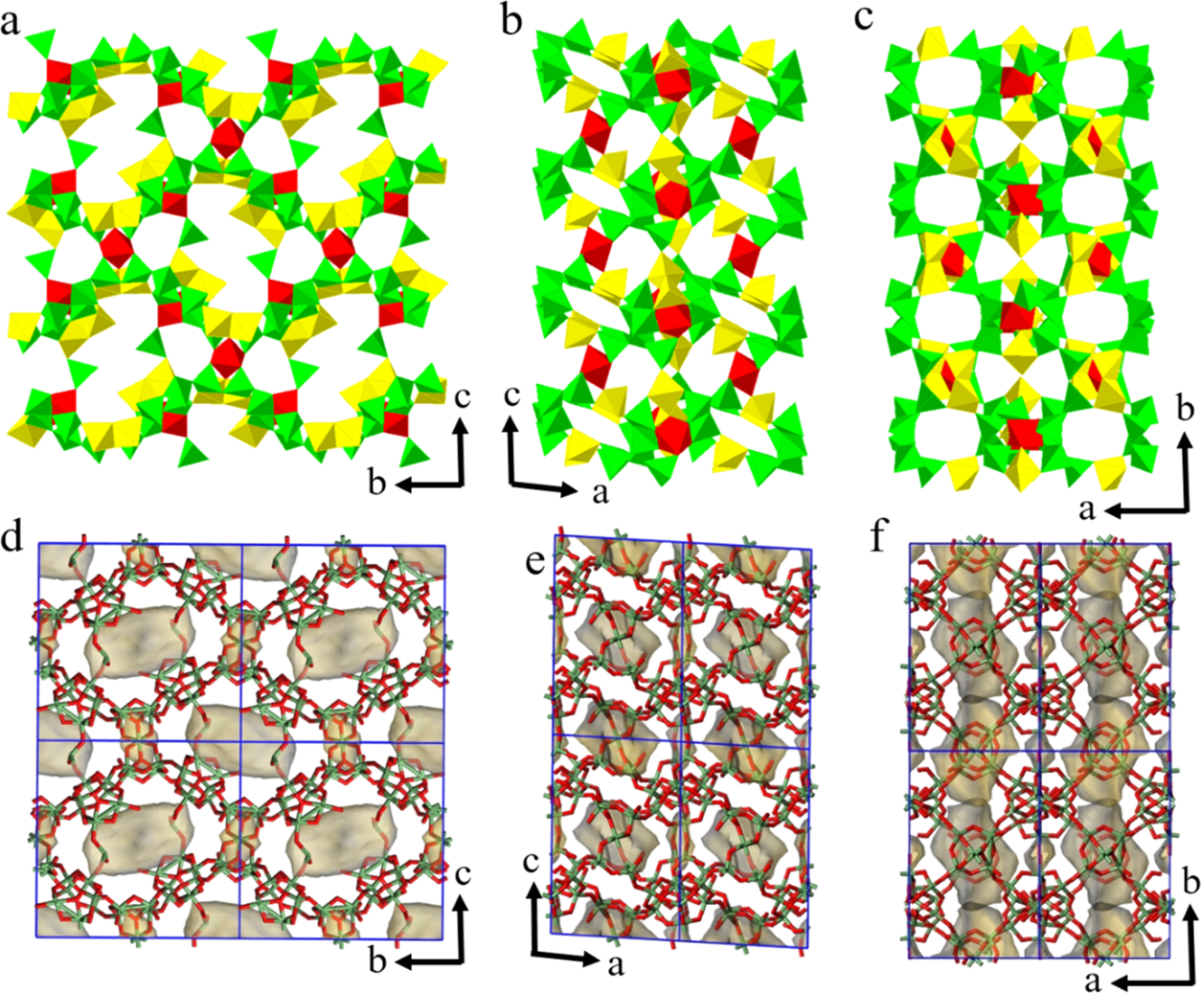
Polyhedral presentation of the framework structure of SU-8 viewed along the (a) *a*-, (b) *b*-, and (c) *c*-axes. Green tetrahedra: GeO_4_ and GeO_2_(OH)_2_, yellow trigonal bipyramids: GeO_5_, and red octahedra: GeO_6_. The accessible pore surface of SU-8 viewed along the (d) *a*-, (e) *b*-, and (f) *c*-axes. The pore surface was calculated using the kinetic diameter of N_2_ (3.64 Å).

**Figure 5:**
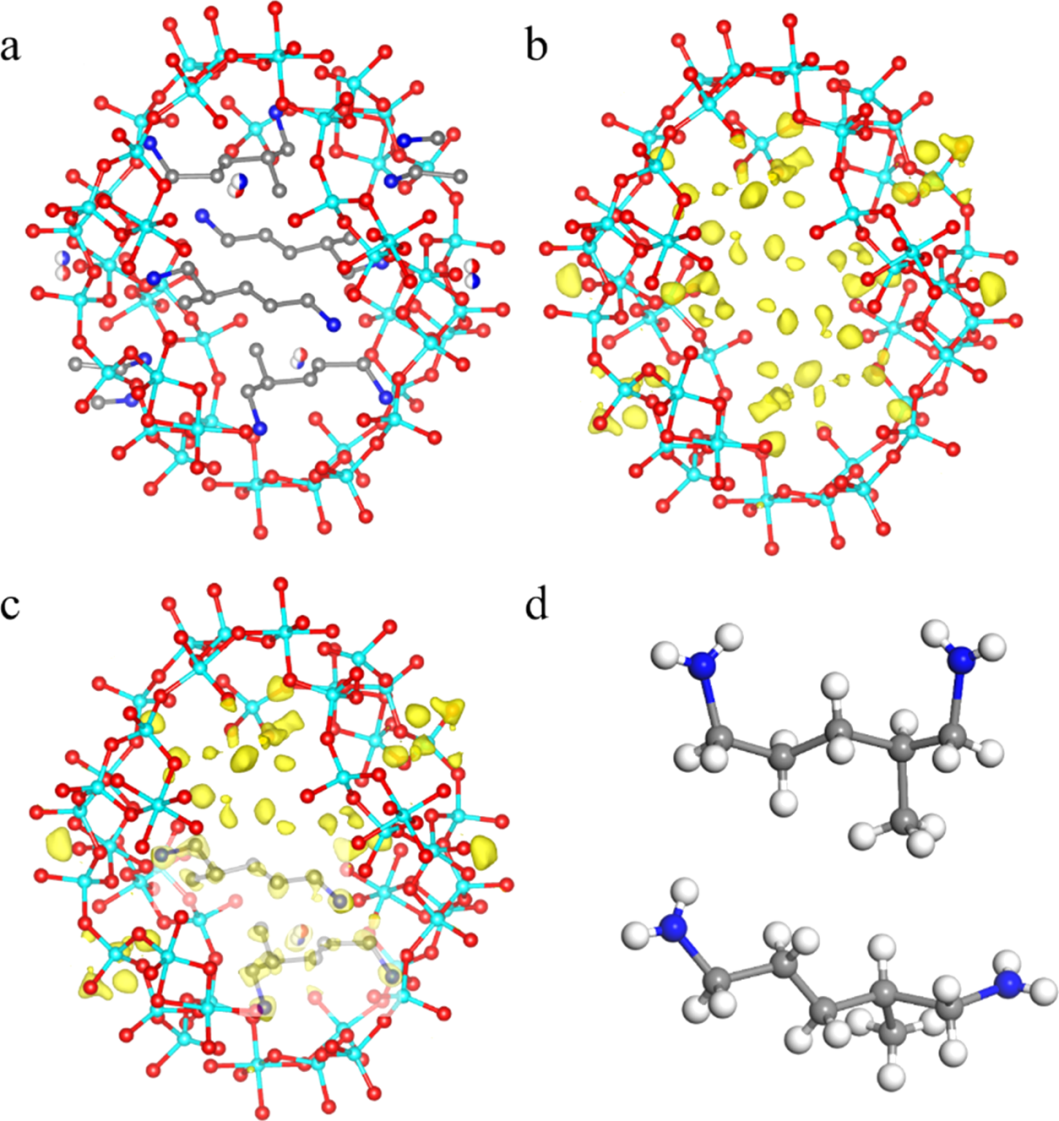
(a) Location of the MPMD molecules in SU-8. (b) Difference electrostatic potential map excluding the guest molecules and (c) with two symmetry-independent MPMD molecules superimposed on the map. The other peaks are generated by symmetry operation. It shows distinct and well-separated peaks, which correspond to the positions of the guest molecules. The electrostatic potential maps are drawn at the 2σ contour level. (d) MPMD molecules showing two spatial configurations inside SU-8. Gray spheres: C, blue spheres: N, cyan spheres: Ge, red spheres: O, and white spheres: H.

The framework of SU-8 is composed of GeO_4_, GeO_2_(OH)_2_, GeO_5_, and GeO_6_ polyhedrons, with 3D interconnected pores that can accommodate guest molecules (Figure 4). Interestingly, in the pores of SU-8, two symmetry-independent MPMD molecules are directly located from structure determination, showing different spatial configurations (Figure 5d). In addition, one O atom from a H_3_O^+^ molecule and one N atom from a disordered MPMD molecule were also located. In total, SU-8 has the following composition [Ge_25_O_60_H_10_]^10–^|(C_6_H_12_N_2_H_6_)_4.5_^2+^(H_3_O)^+^|. To further validate our results that all of the guest molecules are located, we performed refinement without adding guest molecules and calculated the difference electrostatic potential map. It clearly shows distinct and well-separated peaks that are attributed to the guest molecules (Figure 5b,c).

### Comparison of SU-8 by cRED with SCXRD

**Figure 6:**
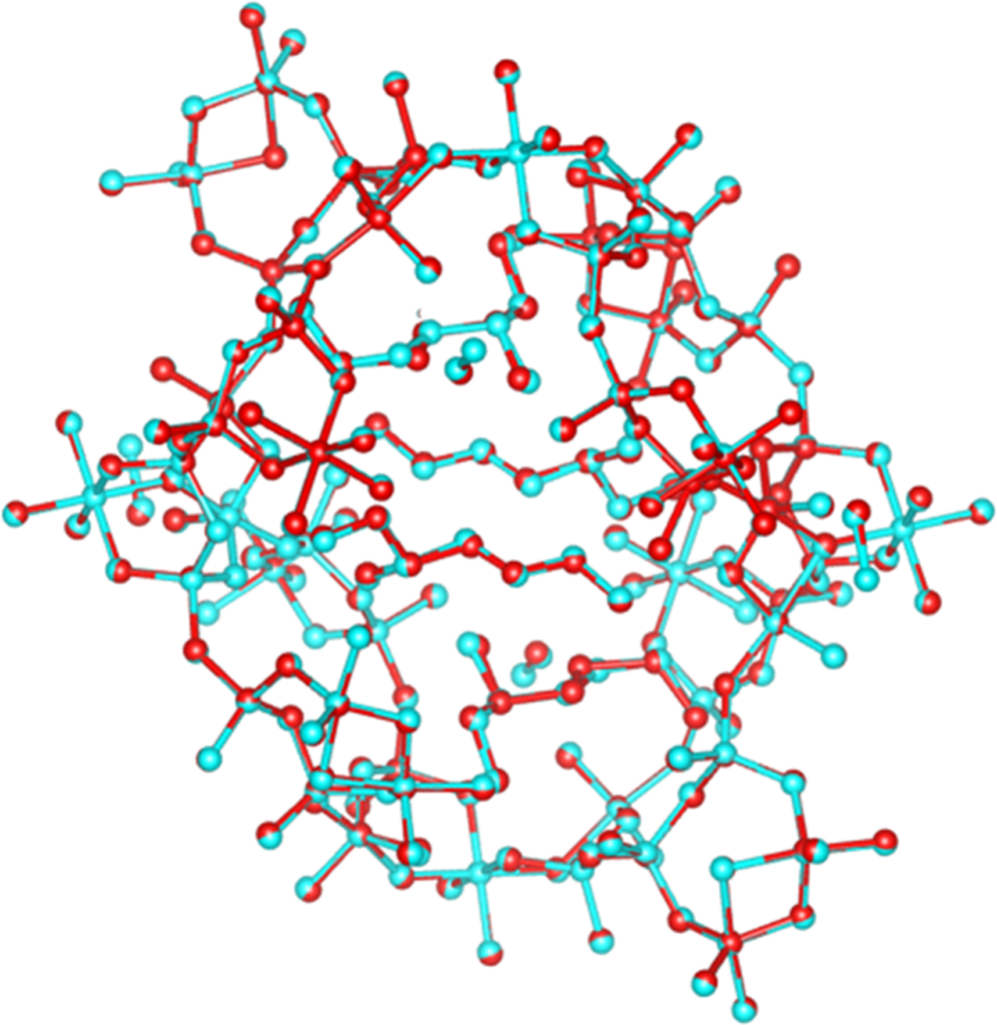
Comparison between the structural models of SU-8 refined from cRED data and SCXRD data. Red: The structural model refined against cRED data; cyan: the structural model refined against SCXRD data.

As the structure of SU-8 has also been determined by SCXRD,^47^ we compared the single-crystal analysis using X-ray data and ED data. With a crystal size 1 000 000 times smaller than that used for SCXRD analysis, cRED data from the nanosized SU-8 shows a higher resolution of 0.74 Å. This emphasizes the advantage to generate high-signal-to-noise data from nanocrystals using electrons. The difference in unit cell parameters determined by SCXRD and cRED is within 3.0% (Table S1). We further investigated the consistency of the models, including the framework structure and guest molecule location obtained using SCXRD and cRED data. We compared the atomic positions of the framework atoms, except for the disordered O 1C and N 1C, belonging to a H_3_O^+^ molecule and a disordered MPMD molecule, respectively. The average deviation is 0.020(4) Å for the heavy Ge atoms and 0.04(2) Å for the O atoms. The atomic positions of the MPMD molecules determined by cRED on average differ by 0.12(6) Å from those determined by SCXRD, within the range of 0.03–0.27 Å (Table S2). The small deviations of Ge and O atoms are due to strong Ge–O bonding and the rigid framework compared to the MPMD molecules, which interact with the framework by weak hydrogen bonding interactions. When the structural models are superimposed, very little difference can be observed (Figure 6). This exhibits excellent agreement between the structural models obtained from SCXRD and cRED, where all nonhydrogen atoms in the organic guest molecules could be precisely located. The relatively large *R*_1_ and Goof values of cRED data could result from the dynamical effects,^37,50^ which are commonly found in 3D ED data, and it could be compensated by the method proposed by Palatinus and co-workers.^51^

### Guest Molecules in Two-Dimensional Framework SU-68

**Figure 7:**
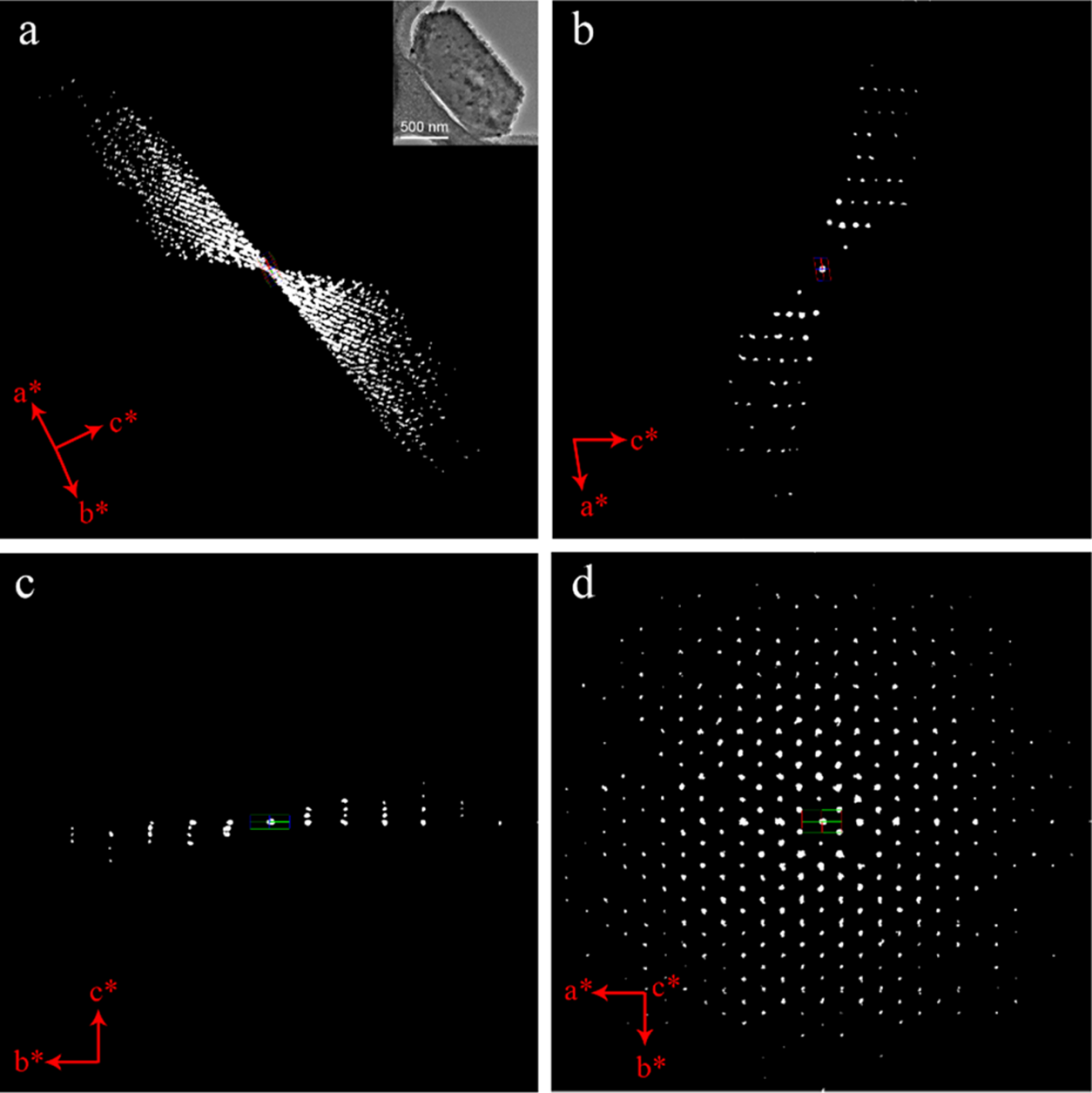
(a) Reconstructed 3D reciprocal lattice of SU-68. The inset shows the crystal morphology of SU-68 (ca. 800 nm in size). 2D slices cut from the reconstructed 3D reciprocal lattice of SU-68 showing the (b) *h*0*l* and (c) 0*kl* planes. (d) 3D reciprocal lattice viewed along the c*-axis. Reflections from ice have been removed for clarity.

SU-68 is a novel Ge-based framework material that was prepared by the hydrothermal method. TAEA was used as an organic molecule to direct the structure, and it remained in the structure thereafter. In our first attempt to collect cRED data from SU-68 nanocrystals, we found that SU-68 can very easily be damaged by an electron beam, which is indicated by a rapid decrease in data resolution (Figure S2c). We, therefore, use a cryo holder to cool the crystal to cryogenic temperature (96 K) and used an ultralow electron dose of 1.0 e^–^ Å^–2^ on average to minimize beam damage. As a result, we were able to acquire cRED data with improved resolution and data coverage (Figure S2b). Compared to SU-8, with little beam damage observed at room temperature (Figure S2a), it indicates that the vulnerability of SU-68 to the electron beam could arise from the stability of the material. By analyzing the cRED data, SU-68 is found to be crystallized in the monoclinic system with a possible space group of **C*2̅*c** (no. 15) and the unit cell parameters of *a* = 14.900(3) Å, *b* = 8.690(2) Å, *c* = 24.730(5) Å, and β = 101.75(3)° (Figures 7 and S3). The cRED data have a lower completeness of SU-68 (76.2%) than that of SU-8 (99.7%), which is due to the strongly preferred orientation of the plate-like SU-68 crystals (Figure 7a inset). Nevertheless, all of the nonhydrogen atoms in the framework and guest molecules can be located directly in a similar way as SU-8 (Table S3).

**Figure 8:**
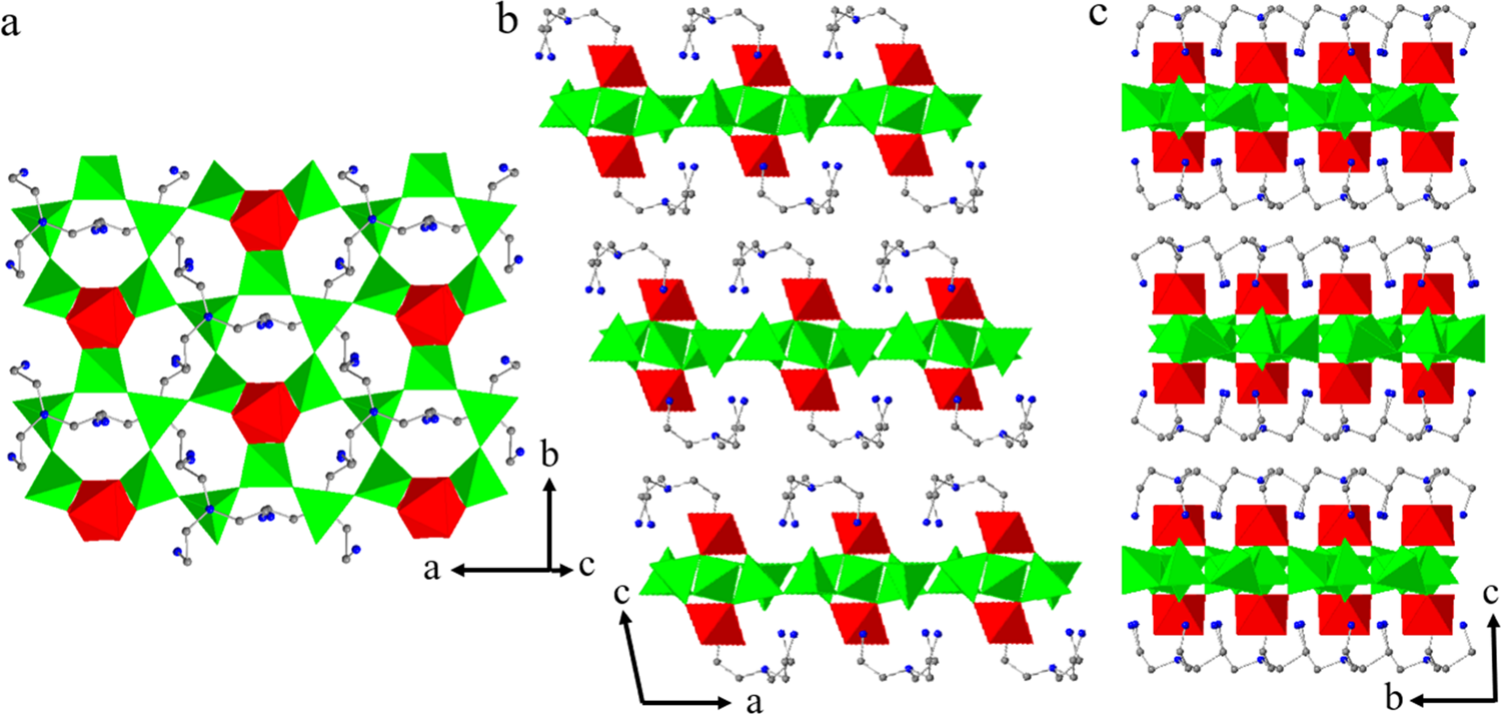
Polyhedral presentation of the framework structure of SU-68 viewed along (a) *c*-axis of one selected layer from the framework structure, (b) *b*-axis, and (c) *a*-axis. Green tetrahedra: GeO_4_, red octahedra: GeO_6_, gray atoms: C, and blue atoms: N.

**Figure 9:**
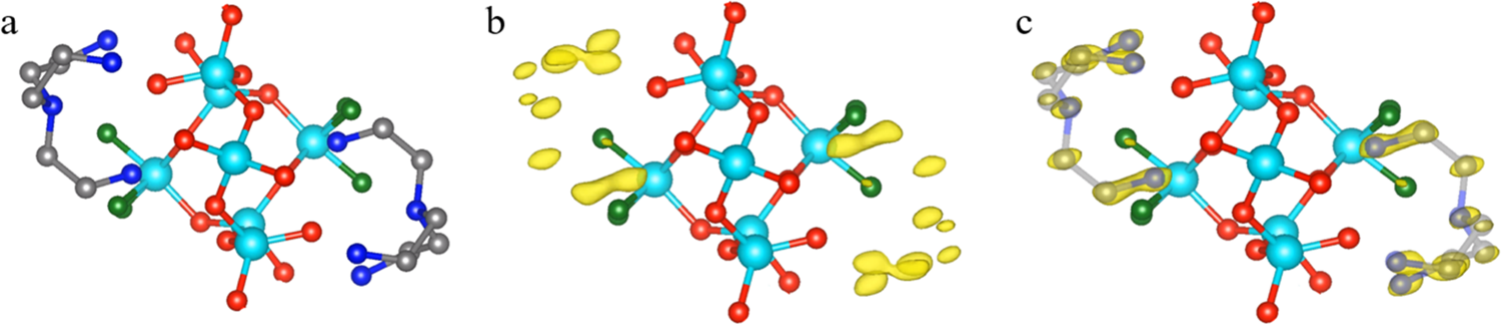
(a) Location of the TAEA molecules in SU-68. The difference electrostatic potential map (b) excluding the guest molecules and (c) with guest molecules superimposed on the map. Gray spheres: C, blue spheres: N, cyan spheres: Ge, red spheres: O, and green spheres: F. The electrostatic potential maps are drawn at the 2σ contour level.

The framework structure of SU-68 was determined to be a 2D layered structure composed of GeO_4_ tetrahedra and GeO_6_ octahedra (Figure 8). The guest molecules of TAEA were found located between the layers, with a total composition of [Ge_7_O_14_(F^–^)_6_]|(N(C_2_H_5_NH_3_^+^)_3_)_2_|. The weight content of the organic molecules agrees well with the TGA result, where the weight loss is due to the combustion of the organics (Figure S4). We investigated the difference electrostatic potential map, and similar to the guest molecules in SU-8, it confirms that all of the atoms of the guest molecules have been located (Figure 9). All of the peaks are well-defined, except for one elongated peak rather than a spherical peak in the difference map. It covers one C and one N atom in the terminal of the TAEA molecule. This could be a result of a relatively low completeness of cRED data of SU-68 compared to that of SU-8 (Figure S5).^52^ To further validate the conformation of organic molecules in SU-68, we have grown large single crystals and performed SCXRD analysis (Table S4). The structures of SU-68, including the frameworks and organic molecules, show a high agreement between those determined from cRED and SCXRD, showing that little difference can be observed from the superimposed structural models (Figure S6).

### Effects of Data Resolution

The advanced 3D ED technique makes it possible to collect a data set in less than 3 min while maintaining an electron dose rate at ∼0.01 e^–^ s^–1^ Å^–2^. This greatly improves the strategies for single-crystal analysis of nanosized framework materials, which typically suffer from electron beam damage. We demonstrate that not only the framework structures but also the location of guest species can be determined using cRED. Nevertheless, the crystallization process could be affected by the kinetics and thermodynamics of the reaction, and result in a different degree of crystallinity for different open-framework materials. Therefore, we investigated the data resolution cutoffs for the potential application of 3D ED to identify guest species in framework materials with lower crystallinity. While we were unable to synthesize the framework materials with varying crystallinities, in our study, we generated resolution-limited data sets by excluding all reflections with *d*-values smaller than a specified resolution value and simulated the intensity/sigma distribution similar to those obtained experimentally (Tables S5 and S6; see the Supporting Information for more details). Structures were solved from each data set using direct methods. On reducing resolution from 0.74 to 1.00 Å of SU-8, all of the atoms can still be directly located despite the slight difference in bond distances and angles from each resolution cutoff (Figure S7a–c). However, when the resolution was cut to 1.10 Å and lower, some atoms started to be missing from the structure solution (circle in Figure S6d and many positions in Figure S7e). For SU-68, reducing resolution from 0.60 to 1.10 Å affects little on locating the TAEA molecules from the structure solution (Figure S8a–f). Further reducing the resolution to 1.20 Å showed a severe distortion of the TAEA molecule (Figure S8g), which could affect its identification. In addition, we performed structural refinement on each data set without including the organic species, and we calculated the corresponding difference electrostatic potential maps. By reducing resolution from 0.74 to 1.00 Å for SU-8 and from 0.60 to 1.00 Å for SU-68, it is still possible to identify the atomic positions of the organic molecules as the peaks in the difference electrostatic potential maps are well defined (Figures S9a,b and S10a–d). However, when resolution is cut below 1.00 Å, there are missing peaks in difference electrostatic potential maps. In addition, with the reduction of data resolution, the peaks tend to evolve from well-distinct peaks to blocks of a large peak. This highlights the importance of using an ultralow dose to obtain high-resolution 3D ED data.

### Host–Guest Interaction

**Figure 10:**
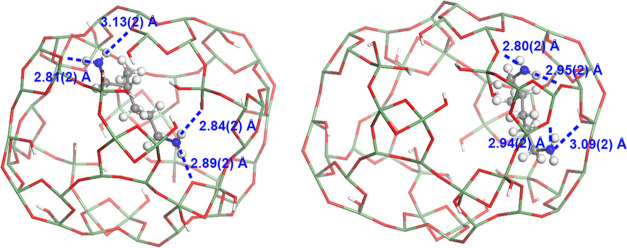
Hydrogen bonds between two symmetry-independent MPMD molecules and the framework of SU-8. Hydrogen bonds (N···O) are represented as dashed blue lines. Green: Ge, red: O, gray: C, blue: N, and white: H.

**Figure 11:**
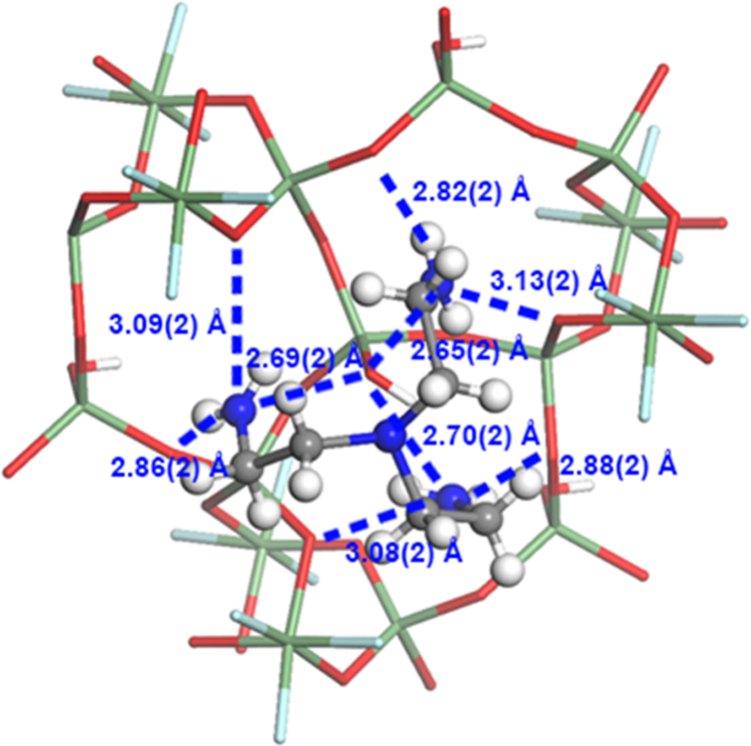
Hydrogen bonds between the TAEA molecule and the framework of SU-68. Hydrogen bonds (N···O) are represented as dashed blue lines. Green: Ge, red: O, gray: C, blue: N, cyan: F, and white: H.

The accurate location of guest molecules allows us to further explore the interactions between the frameworks and guest molecules. In SU-8, the identified guest molecule MPMD consists of two amine groups as terminals at both sides of the molecular chain. Despite different configurations, the two MPMD molecules are immobilized in the pores of SU-8. Both ammonium groups interact with two surrounding O atoms in the framework to form hydrogen bonding (Figure 10 and Table S7), which restricted the molecules to adopt random configurations. This shows that host–guest interactions would be crucial for directly locating guest species by 3D ED. The structural information obtained is averaged over all unit cells. Thus, in the presence of large variation among unit cells, e.g., organic molecules with disorder and low occupancies, accurate location of their positions could be challenging. In SU-68, the organic species TAEA consists of three branches, in which each ammonium group resides as the terminal. While the C–C bond allows free rotation to adopt different spatial configurations of the TAEA molecule, it is found that all terminal ammonium groups point toward the framework of SU-68. Each ammonium group forms hydrogen bonding with three O atoms in the framework (Figure 11 and Table S8). As the ammonium groups in the TAEA molecule bonded to only one layer in SU-68 (Figure 8b,c), it is crucial to understand how the layered structure of SU-68 is stabilized. Looking into details of the organic molecules, weak vdW force can be identified with the closest of 3.76(3) Å. As a result, the layers in SU-68 are connected through the vdW force between the TAEA molecules (Figure 8b,c). However, the weak interaction leads to a relatively unstable framework. Therefore, cooling the crystals during data collection is essential to maintain their structure for a long enough time for acquiring high-quality cRED data and accurately locating the guest molecules. Single-crystal structural analysis requires a high occupancy for atoms to be identified. Thus, 3D ED is advantageous for analyzing guest molecules in nanocrystals because the small size benefits the diffusion process in open-framework materials.

## Conclusions

We show that an ultralow electron dose is not only important for studying biological compounds^53^ but also crucial for studying host–guest interactions in inorganic materials. Using an ultralow electron dose, we demonstrate that the atomic positions of organic molecules in framework materials can be accurately located by applying the 3D ED method at both room temperature and cryogenic temperature. Two framework materials, SU-8 and SU-68, are shown as examples. The structural models of both materials, including their framework structures and organic molecules, are determined ab initio from the cRED data. Notably, from difference electrostatic potential maps, the location of guest molecules can be observed as well-defined peaks, showing high accuracy and reliability. We compared the framework structure of SU-8 and the guest molecule location obtained by SCXRD and cRED, and they show an excellent agreement with an average deviation of 0.020(4) Å for Ge atoms, 0.04(2) Å for O atoms in the framework, and 0.12(6) Å for the atoms in the guest molecule. By cutting the resolution of cRED data, we show that the ab initio location of organic molecules is achievable for resolution as low as 1.00 Å, with the positions of guest molecules being well defined. The location of guest molecules also reveals hydrogen bonding interactions between organic molecules and the frameworks. In addition, organic molecules in SU-68 further interact with each other through vdW force, which stabilizes the 3D structure of SU-68. As many framework materials are synthesized as nanosized crystals and may contain phase mixtures, we foresee that 3D ED will increase its importance in this field. With many questions related to host–guest interactions that have not yet been answered, we believe that 3D ED can provide crucial insights into organic species in other framework materials, such as organic templates in zeolites and guest molecules in MOFs and COFs.

## Supporting Information Available

Additional experimental details and additional results and discussions, including structural analysis by cRED, data simulation for resolution cutoffs, and figures and tables for discussions (PDF)
