# Supplementary material for: Direct Location of Organic Molecules in Framework Materials by Three-Dimensional Electron Diffraction
Source: J Am Chem Soc. 2022 Aug 11;144(33):15165–74. doi: 10.1021/jacs.2c05122 (PMC9434828; doi:10.1021/jacs.2c05122)
Supplement: Supplementary file 1 — ja2c05122_si_001.pdf [file ja2c05122_si_001.pdf]

Supporting Information for

Direct Location of Organic Molecules in Framework Materials by Three-Dimensional Electron Diffraction

Meng Ge[‡], Taimin Yang[‡], Hongyi Xu[‡], Xiaodong Zou[‡,]* and Zhehao Huang[‡,]*

[‡]Department of Materials and Environmental Chemistry, Stockholm University, 106 91 Stockholm, Sweden

Structural analysis by cRED

cRED datasets were collected from several crystals. The best data set was identified according to its large rotation range and apparent good signal-to-noise ratio. For SU-8, a typical cRED data contains 740 electron diffraction frames, collected from a crystal of about 1.0 μm in size at a tilting angle of 116.40° at a tilting speed of 0.45° s^{-1}. The total data collection time was 4.3 min. For SU-68, a typical cRED collected at 96 K contains 185 electron diffraction frames, collected from a crystal of about 600 nm in size in a tilting angle of 47.03° at a tilting speed of 0.45° s^{-1}. The total data collection time was 1.7 min. In total, 7 datasets of SU-8 and SU-68, respectively, were analyzed and merged for structure determination. To obtain reliable data with high resolutions, the detector was calibrated by using gold nanoparticles under different camera lengths. For SU-8, data was collected using a camera length of 25 cm, which corresponding to a scale of 0.00527 $Å^{-1}$ per pixel. For SU-68, data was collected using a camera length of 20 cm, which corresponding to a scale of 0.00658 $Å^{-1}$ per pixel.

We use the *REDprocessing* software[1] for the initial unit cell and space group determination and visualization of 3D reciprocal lattice. For SU-8, The unit cell parameters were determined to be a = 12.21 Å, b = 19.38 Å, c = 18.55 Å, α = 90.27°, β = 92.14°, γ = 90.70°. The intensity distribution of reflections in the 3D reciprocal lattice indicates that the crystal is monoclinic with the Laue class 2/m. The unit cell angles α, and γ is near 90°, which also confirms the monoclinic crystal system. The reflection conditions can be deduced from the two-dimensional (2D) slice cut as $0k0$: $k=2n$; $00l$: $l=2n$ and $h0l$: $l=2n$ (Figures 3a-c). This corresponds to the space group of $P2_1/c$ (No. 14). For SU-68, the unit cell parameters were determined to be a = 14.96 Å, b = 8.57 Å, c = 24.16

Å, α = 89.15°, β = 102.49°, γ = 88.85°. The intensity distribution of reflections in the 3D reciprocal lattice indicates that the crystal is monoclinic with the Laue class 2/*m*. The unit cell angles α, and γ are near 90°, which also confirms the monoclinic crystal system. The reflection conditions can be deduced from the two-dimensional (2D) slice cut as *h*0*l*: *l*=2*n*, *h*=2*n*; 0*k*0: *k*=2*n* and *hkl*: *h*+*k*=2*n* (Figures 7a-c), which correspond to the possible space group of *Cc* (No. 9) and *C*2/*c* (No. 15). The higher symmetry space group *C*2/*c* was used for further analysis.

The intensities of the reflections were extracted from the cRED data using the X-ray Detector Software (*XDS*)[2]. To achieve ultralow dose, we collected data at a tilting range as small as 35°. Therefore, data merging is important and essential for further analysis. We use *XSCALE* for data merging, and we access the quality of data using correlation coefficients of the common reflection intensities (CC_I)[3]. To achieve high data quality, we only include datasets with high CC_I (> 0.90) for merging. The cRED data of SU-8 reached a resolution of 0.74 Å and a completeness of 99.7% after merging seven cRED datasets. Assuming the direct beam is placed at the center of the detector, the camera length of 20 cm gives the maximum resolution of 0.420 Å to the corner of the detector and 0.594 Å to the edge of the detector. As the longest axis of SU-68 is 24.730(5) Å, which corresponding to a vector of 0.0404 $Å^{-1}$ in the reciprocal space. Thus, 6.14 pixels separate the closest reflection pairs of SU-68 when using the camera length of 20 cm. As a result, XDS can reliably index the reflections with a resolution of 0.60 Å for SU-68. The completeness of SU-68 is 76.2% by merging seven datasets. The positions of all non-hydrogen atoms were found directly from the structure solution by direct methods using the SHELX software package[4]. For the refinement of the merged dataset of SU-8, atomic anisotropic displacement parameters were applied on all Ge atoms. The final refinement converged with the agreement values R_1=0.220 for 10101 reflection with $F_o > 4\sigma(F_o)$ and 0.235 for all 11776 reflections. For the refinement of the merged dataset of SU-68, atomic anisotropic displacement parameters were applied on all the framework atoms including Ge and O. The final refinement converged with the agreement values R_1=0.260 for 4903 reflections with $F_o > 4\sigma(F_o)$ and 0.297 for all 5795 data. The relatively large R_1 value resulted from the dynamical effects[5].

Data simulation for resolution cutoffs

We use the Computational Crystallography Toolbox (CCTBX)[6] library to simulate date in similar conditions as reduced crystallinity. We first cut the resolution, by excluding all reflections with d-values smaller than a specified resolution value. The data with different resolution cutoffs are divided into the same number of resolution shells with similar numbers of reflections in each shell, i.e., 18 resolution shells for SU-8, and 17 resolution shells for SU-68. Then, we processed the hkl files and simulated sigma values in hkl files using Python CCTBX library so that the distribution of I/sigma at each resolution shell matches with the distribution of that before the resolution cut (Tables S5 and S6).

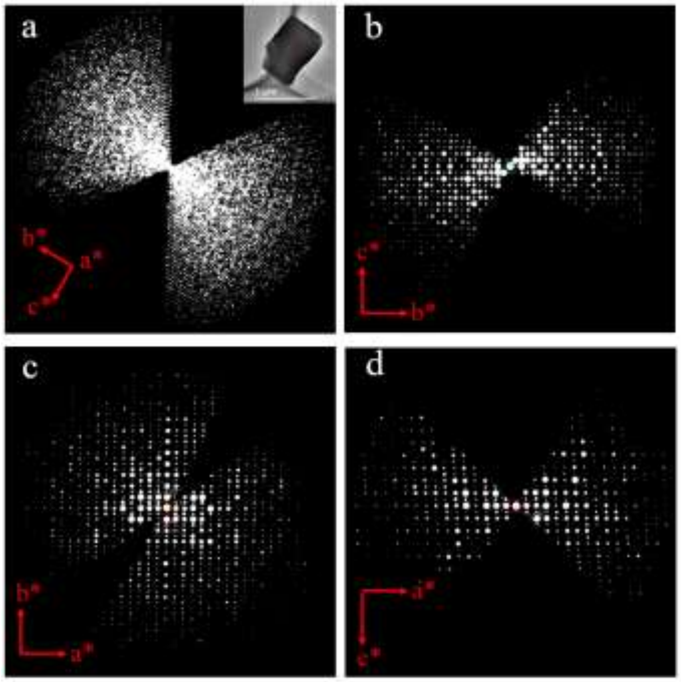

Figure S1. (a) Reconstructed 3D reciprocal lattice of SU-8. Inset shows the crystal morphology of SU-8 (ca. 900 nm in size). 2D slice cuts from the reconstructed 3D reciprocal lattice of SU-8 showing the (b) 0*kl*, and (c) *hk*0, and (d) *h*0*l* planes.

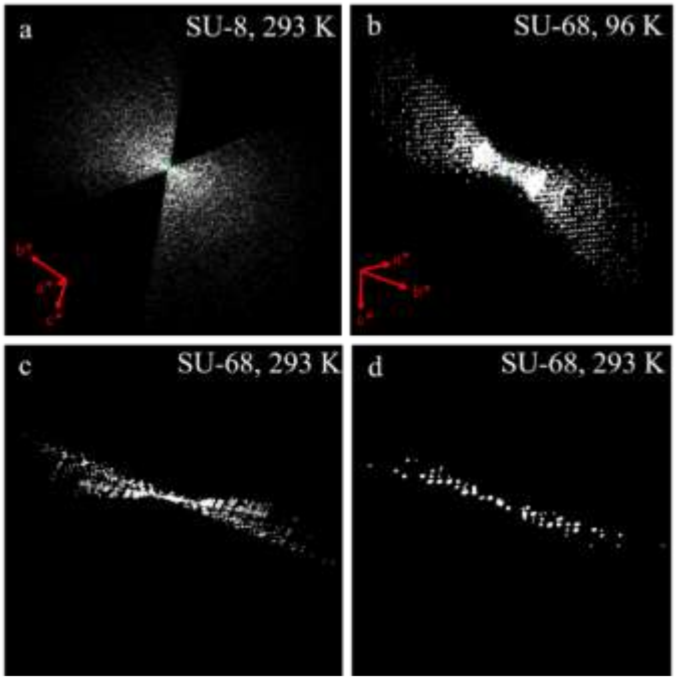

Figure S2. Reconstructed 3D reciprocal lattice of (a) the SU-8 crystal collected at room temperature (293 K), (b) the SU-68 crystal collected at 96 K, and (c and d) two SU-68 crystals collected at room temperature (293 K). Collecting SU-68 data at room temperature shows a rapid decrease on data resolution, and diffraction spots can only be observed in a small tilting range, beyond which the crystals become completely amorphous.

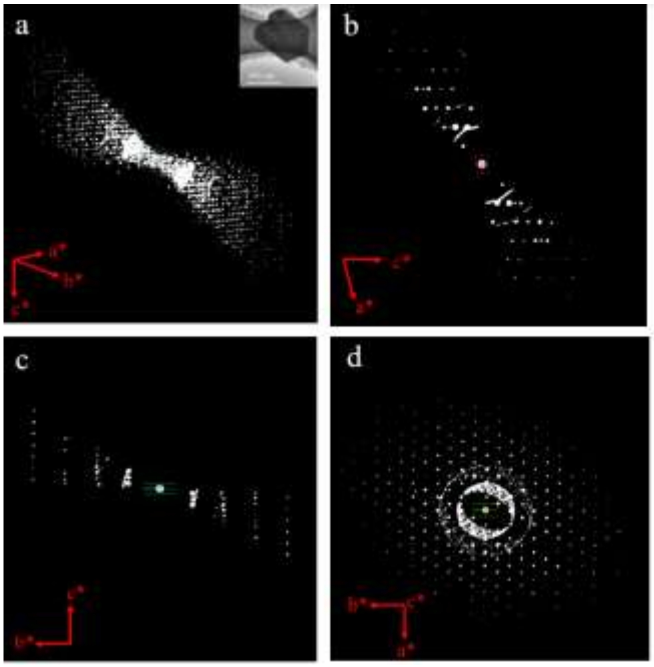

Figure S3. (a) Reconstructed 3D reciprocal lattice of SU-68. Inset shows the crystal morphology of SU-68 (ca. 600 nm in size). 2D slices cut from the reconstructed 3D reciprocal lattice of SU-68 showing the (b) *h*0*l* and (c) 0*kl* planes. (d) 3D reciprocal lattice viewed along *c**-axis. Diffused scattering from ice can be observed in the background.

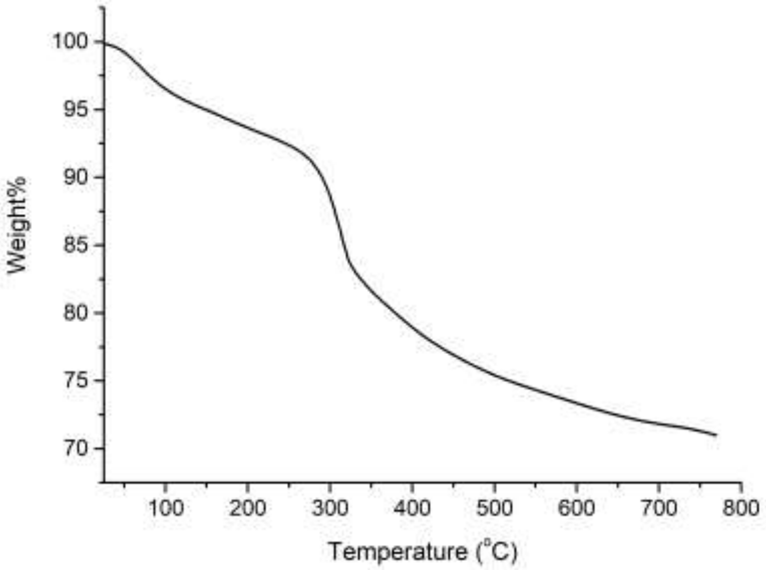

Figure S4. Thermogravimetric analysis of SU-68 under air flow. The weight loss is due to the combustion of organic molecules. The total weight loss is 28%, which agrees well with 26% weight of organic molecules in SU-68 from the chemical formula concluded by SCXRD and cRED.

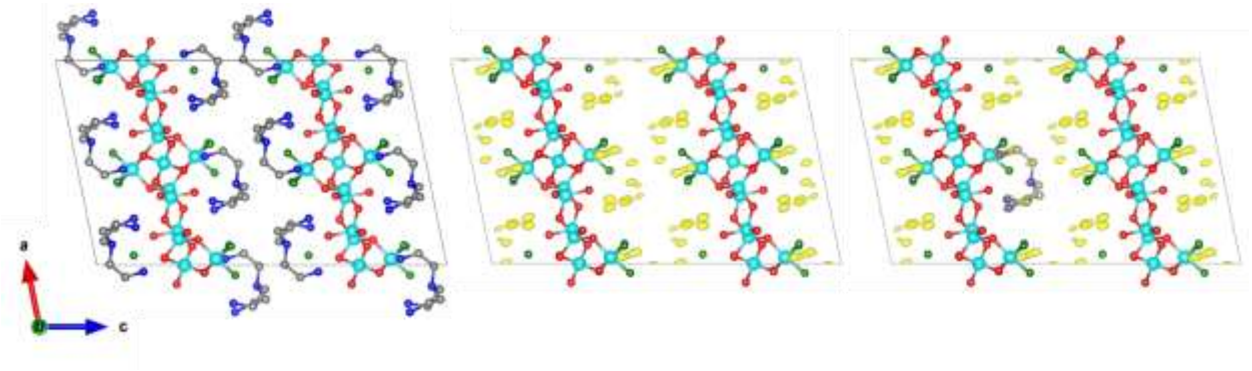

Figure S5. Location of the TAEA molecules in SU-68. The difference electrostatic potential map shows one elongated peak covering one C and one N atoms. The elongation is along the *c*-axis, where the data has the most missing wedge. Grey spheres: C; blue spheres: N; cyan spheres: Ge; red spheres: O; green spheres: F. The electrostatic potential maps are drawn at a 2σ contour level.

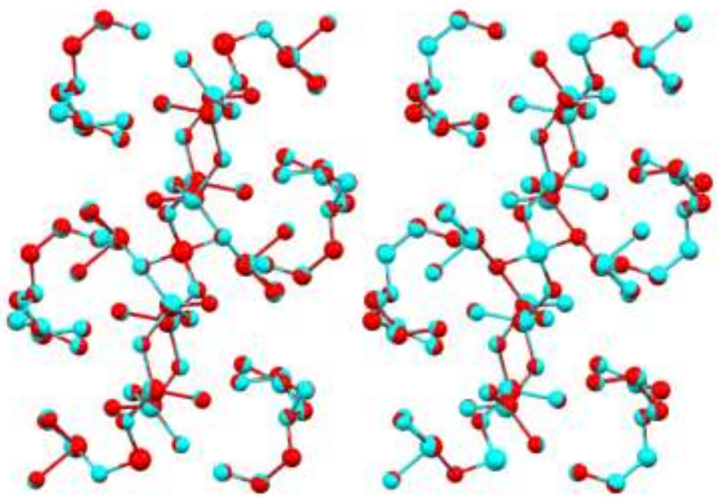

Figure S6. Comparison between the structural models of SU-68 determined from cRED data and SCXRD data. Red: The structural model refined against cRED data; cyan: the structural model refined against SCXRD data.

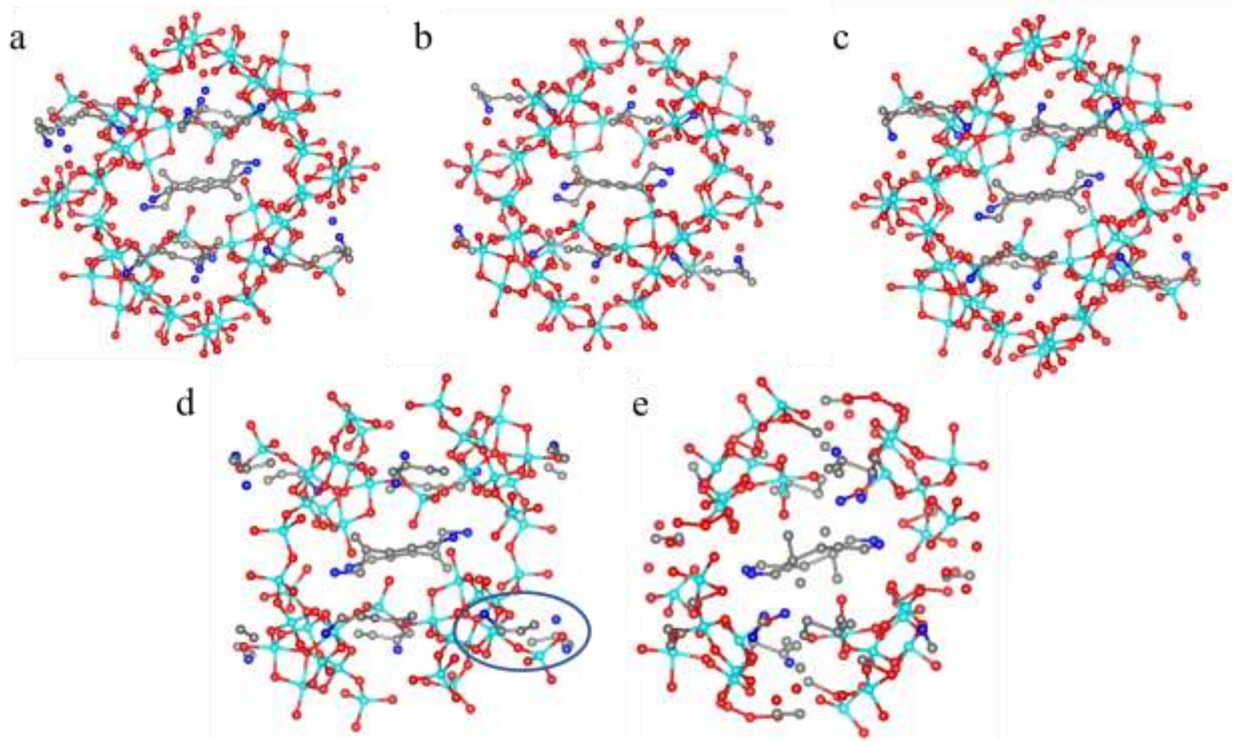

Figure S7. Direct location of the MPMD molecules in SU-8 from structure solution of the data (a) without cutting resolution, (b-e) with the resolution cutoff of 0.9 Å, 1.0 Å, 1.1 Å, and 1.2 Å, respectively. Grey spheres: C; blue spheres: N; cyan spheres: Ge; red spheres: O.

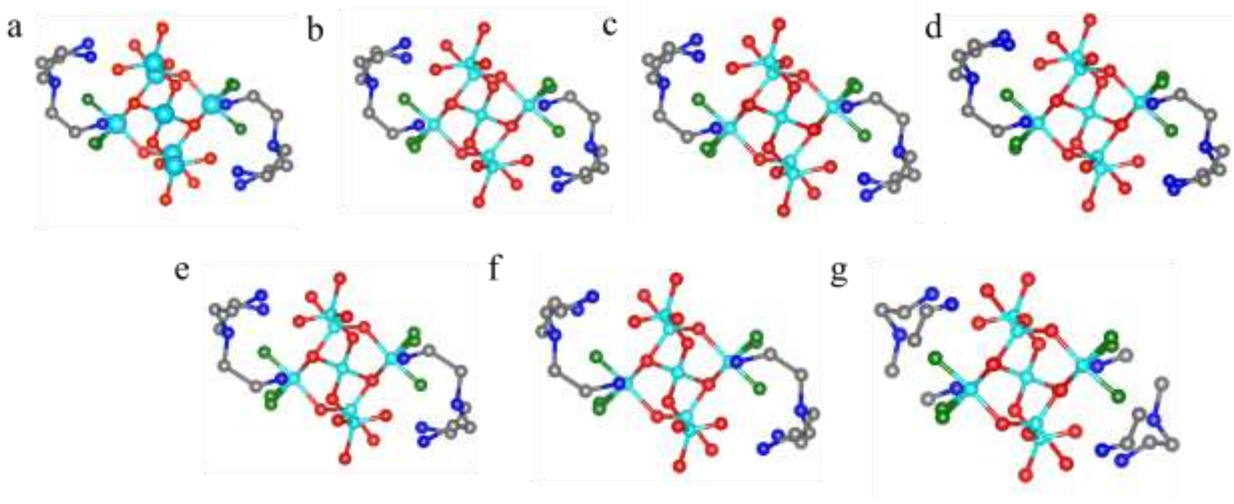

Figure S8. Direct location of the TAEA molecules in SU-68 from structure solution of the data (a) without cutting resolution, (b-g) with the resolution cutoff of 0.7 Å, 0.8 Å, 0.9 Å, 1.0 Å, 1.1 Å, and 1.2 Å, respectively. Grey spheres: C; blue spheres: N; cyan spheres: Ge; red spheres: O; yellow spheres: F.

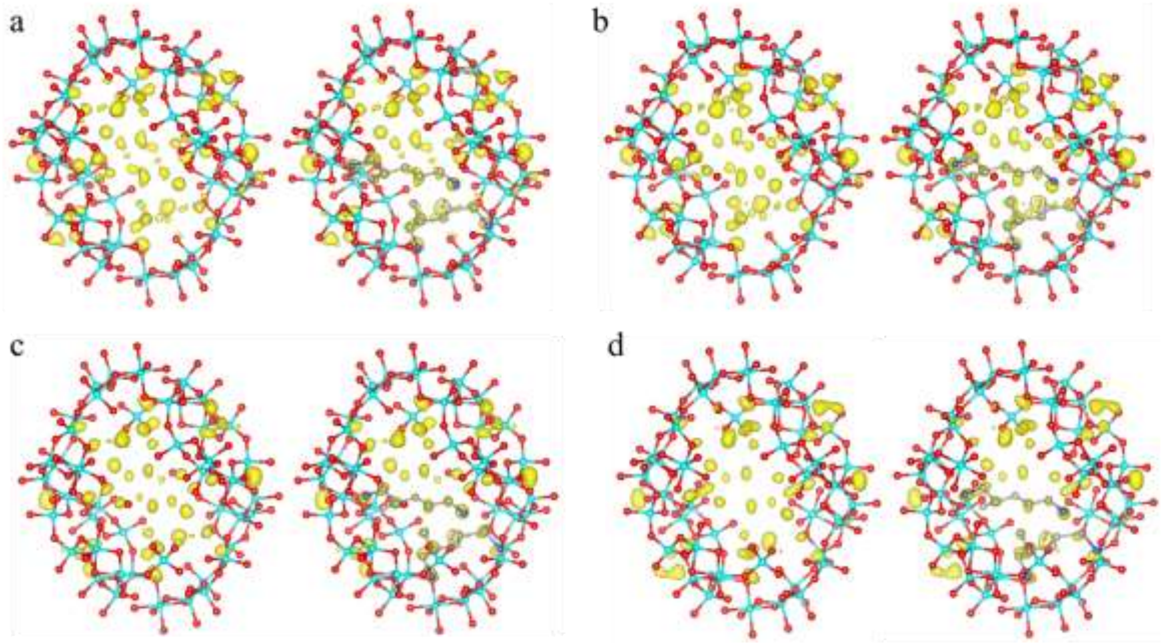

Figure S9. The difference electrostatic potential maps of SU-8 calculated from the data with the resolution cutoff of (a) 0.9 Å, (b) 1.0 Å, (c) 1.1 Å, and (d) 1.2 Å. The electrostatic potential maps are drawn at the same isosurface level of d_{iso} = 0.34. Cyan: Ge; red: O.

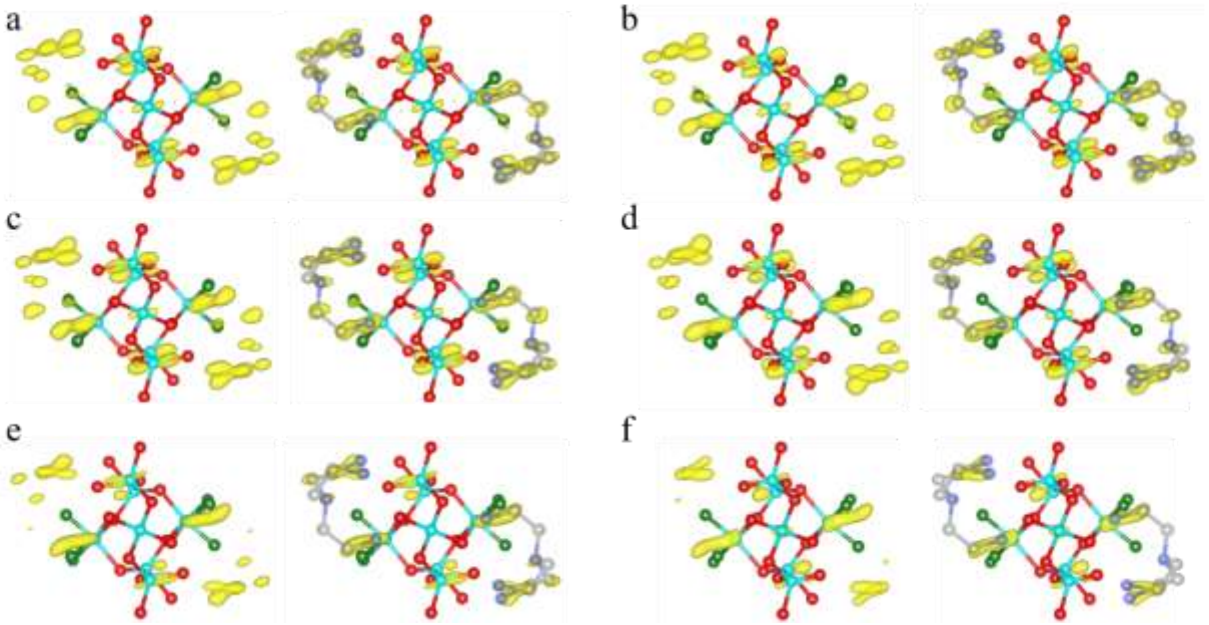

Figure S10. The difference electrostatic potential maps of SU-68 calculated from the data with the resolution cutoff of (a) 0.7 Å, (b) 0.8 Å, (c) 0.9 Å, (d) 1.0 Å, (e) 1.1 Å, and (f) 1.2 Å. The electrostatic potential maps are drawn at the same isosurface level of d_{iso} = 0.55. Cyan: Ge; red: O.

Table S1. Comparison of crystallographic data and refinement details of SU-8 refined against cRED and SCXRD data.

	cRED data	SCXRD data[7]
No. dataset merged	7	1
Wavelength (Å)	0.0251 Å	0.71073 Å
Crystal size (μm^3)	0.6 × 0.7 × 1.0	50 × 60 × 150
Average electron dose (e$^-$ Å^{-2})	2.4	-
Resolution (Å)	0.74	0.79
Crystal system	Monoclinic	Monoclinic
Space group	$P2_1/c$ (No. 14)	$P2_1/c$ (No. 14)
Unit cell *a*, *b*, *c* (Å), *β* (°)	12.169(2), 19.442(4), 19.289(4), 92.45(3)	12.075(4), 19.235(6), 18.720(6), 92.72(4)
Volume (Å^3)	4559(2)	4343(2)
Completeness (%)	99.7	98.3
No. unique reflections	11776	8327
No. observed reflections (I > 2 sigma(I))	10101	5238
No. refined parameters/restraints	311/1	307/1
R_1 (I > 2 sigma(I))	0.220	0.048
R_1 (all reflections)	0.235	0.095
Goof	1.009	1.037

Table S2. Comparison of the atomic positions of SU-8 refined against cRED data with those refined against SCXRD data[7].

*Atom**	*x, y, z coordinates from cRED*			*x, y, z coordinates from SCXRD*			*Difference (Å)*
Ge1	0.5000	0.5000	1.0000	0.5000	0.5000	1.0000	0
Ge2	-0.3899(3)	0.5584(2)	0.8342(2)	-0.39198(8)	0.55847(4)	0.83418(5)	0.025(4)
Ge3	-0.3738(3)	0.4083(2)	0.8516(2)	-0.37381(8)	0.40814(5)	0.85100(5)	0.012(4)
Ge4	0.3802(3)	0.40453(18)	0.89110(19)	0.38024(7)	0.40434(4)	0.89188(5)	0.015(4)
Ge5	0.3642(3)	0.55947(19)	0.87234(19)	0.36320(8)	0.55958(4)	0.87220(5)	0.012(4)
Ge6	0.0295(3)	0.25767(19)	0.71945(18)	0.03061(8)	0.25878(4)	0.71998(5)	0.027(4)
Ge7	-0.0285(3)	0.21725(19)	0.90718(19)	-0.02854(7)	0.21655(4)	0.90713(5)	0.014(4)
Ge8	-0.0009(3)	0.0952(2)	0.82026(19)	-0.00108(8)	0.09437(5)	0.81961(5)	0.020(4)
Ge9	0.2215(3)	0.1527(2)	0.76116(19)	0.22285(7)	0.15306(4)	0.76095(5)	0.018(4)
Ge10	-0.1682(3)	0.15423(19)	0.7072(2)	-0.16754(7)	0.15520(4)	0.70764(5)	0.022(4)
Ge11	-0.1975(3)	0.2856(2)	0.79842(19)	-0.19720(8)	0.28629(5)	0.79838(5)	0.014(4)
Ge12	0.1885(3)	0.28181(18)	0.85162(19)	0.18938(7)	0.28134(4)	0.85336(5)	0.035(4)
Ge13	-0.0398(3)	0.33537(19)	0.57418(19)	-0.03778(8)	0.33689(5)	0.57511(5)	0.041(4)
Average Ge atoms							0.020(4)
O1	0.5947(9)	0.5767(6)	1.0198(6)	0.5948(5)	0.5752(3)	1.0191(3)	0.03(1)
O2	0.3884(8)	0.5605(5)	0.9629(5)	0.3891(5)	0.5606(3)	0.9640(3)	0.02(1)
O3	0.4483(9)	0.5089(5)	1.0943(5)	0.4485(5)	0.5101(3)	1.0957(3)	0.03(1)
O4	-0.3065(13)	0.5967(8)	0.8997(8)	-0.3029(5)	0.5987(3)	0.8992(4)	0.06(2)
O5	-0.5290(8)	0.5921(5)	0.8202(5)	-0.5304(5)	0.5915(3)	0.8202(3)	0.02(1)
O6	-0.3542(9)	0.4800(6)	0.7930(5)	-0.3544(5)	0.4790(3)	0.7909(3)	0.04(1)

O7	0.3457(10)	0.1143(6)	0.7391(6)	0.3465(5)	0.1141(3)	0.7399(3)	0.02(1)
O8	-0.2868(13)	0.3952(9)	0.9264(8)	-0.2797(5)	0.3982(3)	0.9255(3)	0.11(2)
O9	0.4904(9)	0.3661(6)	0.8474(6)	0.4926(5)	0.3665(3)	0.8486(3)	0.03(1)
O10	-0.3117(9)	0.3416(6)	0.7947(6)	-0.3119(5)	0.3405(3)	0.7964(3)	0.04(1)
O11	0.2768(10)	0.3415(6)	0.8934(6)	0.2779(5)	0.3414(3)	0.8951(3)	0.03(1)
O12	0.3230(9)	0.4758(6)	0.8434(5)	0.3211(5)	0.4758(3)	0.8427(3)	0.03(1)
O13	-0.2537(10)	0.1176(6)	0.6388(6)	-0.2541(5)	0.1198(3)	0.6391(3)	0.04(1)
O14	0.0551(9)	0.3124(6)	0.6413(5)	0.0584(5)	0.3127(3)	0.6413(3)	0.04(1)
O15	0.1237(9)	0.3160(6)	0.7768(6)	0.1252(5)	0.3152(3)	0.7773(3)	0.03(1)
O16	-0.0687(9)	0.2023(6)	0.6647(6)	-0.0676(5)	0.2021(3)	0.6649(3)	0.01(1)
O17	0.1483(10)	0.2005(6)	0.6957(6)	0.1496(5)	0.2010(3)	0.6965(3)	0.02(1)
O18	-0.0947(8)	0.3134(5)	0.7436(5)	-0.0929(5)	0.3158(3)	0.7442(3)	0.05(1)
O19	-0.0008(9)	0.1984(5)	0.8063(5)	0.0016(5)	0.1984(3)	0.8057(3)	0.03(1)
O20	0.0977(9)	0.2633(6)	0.9188(6)	0.0983(5)	0.2624(3)	0.9225(3)	0.07(1)
O21	-0.0301(9)	0.1256(6)	0.9072(5)	-0.0313(5)	0.1242(3)	0.9078(3)	0.03(1)
O22	-0.1526(10)	0.2674(6)	0.8850(6)	-0.1499(5)	0.2671(3)	0.8857(3)	0.04(1)
O23	-0.0573(9)	0.2768(5)	0.5037(5)	-0.0578(5)	0.2785(3)	0.5040(3)	0.03(1)
O24	-0.1131(10)	0.0848(6)	0.7556(6)	-0.1146(5)	0.0845(3)	0.7571(3)	0.03(1)
O25	0.1359(9)	0.0882(6)	0.7938(6)	0.1386(5)	0.0866(3)	0.7924(3)	0.05(1)
O26	-0.0060(10)	0.0040(7)	0.8422(6)	-0.0031(5)	0.0021(3)	0.8420(3)	0.05(1)
O27	0.2689(10)	0.2094(6)	0.8309(6)	0.2687(5)	0.2086(3)	0.8328(3)	0.04(1)
O28	-0.2524(10)	0.2067(6)	0.7609(6)	-0.2548(5)	0.2084(3)	0.7587(3)	0.06(1)
O29	0.0016(12)	0.4157(7)	0.5405(7)	0.0048(5)	0.4176(3)	0.5412(3)	0.05(2)
O30	-0.1726(12)	0.3453(7)	0.6111(7)	-0.1690(5)	0.3468(3)	0.6138(3)	0.07(1)
Average O atoms							0.04(2)
N1A	0.1979(12)	0.0640(8)	0.2114(7)	0.1900(7)	0.0634(4)	0.2097(4)	0.10(2)
C1A	0.257(2)	0.0702(13)	0.1483(12)	0.2597(10)	0.0716(6)	0.1453(6)	0.07(3)
C2A	0.280(3)	0.0030(15)	0.1042(14)	0.2836(11)	0.0004(7)	0.1079(7)	0.09(3)
C3A	-0.329(3)	0.516(2)	0.4675(17)	-0.3420(16)	0.5158(9)	0.4639(10)	0.17(4)
C4A	-0.269(2)	0.5010(15)	0.5261(13)	-0.2682(11)	0.4980(6)	0.5284(7)	0.07(4)
C5A	-0.324(2)	0.4995(14)	0.5971(11)	-0.3305(9)	0.5019(5)	0.5973(6)	0.09(3)
C6A	0.358(3)	-0.039(2)	0.1573(18)	0.3565(14)	-0.0463(8)	0.1552(9)	0.15(4)
N2A	-0.2580(15)	0.4737(8)	0.6569(9)	-0.2585(7)	0.4739(4)	0.6584(4)	0.03(2)
N1B	-0.2644(15)	0.2154(9)	0.5181(9)	-0.2592(7)	0.2142(4)	0.5139(4)	0.11(2)
C1B	-0.363(3)	0.266(2)	0.489(2)	-0.3468(12)	0.2661(7)	0.4883(8)	0.20(4)
C2B	-0.458(3)	0.2528(19)	0.5193(15)	-0.4630(13)	0.2526(7)	0.5138(8)	0.12(3)
C3B	-0.449(3)	0.2728(19)	0.5977(16)	-0.4557(12)	0.2697(7)	0.5968(8)	0.10(4)
C4B	-0.559(4)	0.273(4)	0.629(3)	-0.5711(17)	0.2617(10)	0.6313(11)	0.27(7)
C5B	-0.562(2)	0.2823(15)	0.7037(13)	-0.5656(10)	0.2804(6)	0.7072(6)	0.09(3)
C6B	-0.547(4)	0.298(2)	0.480(2)	-0.5422(13)	0.3013(8)	0.4719(9)	0.18(4)
N2B	-0.4947(17)	0.2325(11)	0.7542(11)	-0.5012(7)	0.2305(4)	0.7564(5)	0.10(2)
Average MPMD							0.12(6)

*The positions of O1C and N1C were not included in the comparison as O1C was determined as a H_3O^+ molecule, and N1C was determined as part of a disordered MPMD molecule.

Table S3. Crystallographic data and refinement details of SU-68 by cRED.

	cRED data
No. dataset merged	7
Wavelength (Å)	0.02508 Å
Crystal size (μm^3)	0.6 × 0.7 × 0.6
Average electron dose (e$^-$ Å^{-2})	1.0
Resolution (Å)	0.60
Crystal system	Monoclinic
Space group	*C*2/*c* (No. 15)
Unit cell *a*, *b*, *c* (Å), *β* (°)	14.900(3), 8.690(2), 24.730(5), 101.75(3)
Volume (Å^3)	3135(1)
Completeness (%)	76.2
No. unique reflections	5795
No. observed reflections (I > 2 sigma(I))	4903
No. refined parameters/restraints	113/0
R_1 (I > 2 sigma(I))	0.260
R_1 (all reflections)	0.297
Goof	2.695

Table S4. Crystallographic data and refinement details of SU-68 by SCXRD.

	SCXRD data
Wavelength (Å)	0.71073 Å
Resolution (Å)	0.80
Crystal system	Monoclinic
Space group	*C*2/*c* (No. 15)
Unit cell *a*, *b*, *c* (Å), *β* (°)	14.7405(4), 8.5108(2), 24.7233(8), 101.453(3)
Volume (Å^3)	3039.9(2)
Completeness (%)	99.3
No. unique reflections	3117
No. observed reflections (I > 2 sigma(I))	1551
No. refined parameters/restraints	213/0
R_1 (I > 2 sigma(I))	0.037
R_1 (all reflections)	0.097
Goof	0.730

Table S5. Comparison of data statistics of SU-8 before and after resolution cutoff and simulation.

**0.74 Å (original)**		**0.90 Å**		**1.00 Å**	
**Resolution**	**I/sigma**	**Resolution**	**I/sigma**	**Resolution**	**I/sigma**
Inf - 2.01	7.77	Inf - 2.44	7.77	Inf - 2.72	7.77
2.01 - 1.60	7.50	2.44 - 1.94	7.50	2.72 - 2.15	7.51

1.60 - 1.40	7.18	1.94 - 1.70	7.49	2.15 - 1.88	7.48
1.40 - 1.27	7.12	1.70 - 1.54	7.19	1.88 - 1.71	7.19
1.27 - 1.18	6.67	1.54 - 1.43	7.09	1.71 - 1.59	7.12
1.18 - 1.11	5.99	1.43 - 1.34	6.63	1.59 - 1.49	6.60
1.11 - 1.05	5.72	1.34 - 1.27	6.02	1.49 - 1.41	6.01
1.05 - 1.00	5.64	1.27 - 1.21	6.01	1.41 - 1.35	5.98
1.00 - 0.96	5.47	1.21 - 1.16	6.48	1.35 - 1.29	6.46
0.96 - 0.92	5.10	1.16 - 1.12	5.41	1.29 - 1.24	5.48
0.92 - 0.89	4.85	1.12 - 1.08	5.09	1.24 - 1.20	5.14
0.89 - 0.86	4.55	1.08 - 1.05	4.77	1.20 - 1.16	4.88
0.86 - 0.83	4.01	1.05 - 1.02	4.37	1.16 - 1.13	4.39
0.83 - 0.81	3.62	1.02 - 0.99	3.97	1.13 - 1.10	3.92
0.81 - 0.79	3.22	0.99 - 0.96	3.55	1.10 - 1.07	3.53
0.79 - 0.77	2.66	0.96 - 0.94	3.00	1.07 - 1.05	2.95
0.77 - 0.75	2.32	0.94 - 0.92	2.39	1.05 - 1.02	2.46
0.75 - 0.74	2.01	0.92 - 0.90	2.11	1.02 - 1.00	2.12
**Overall**	5.31	**Overall**	5.33	**Overall**	5.34

**1.10 Å**		**1.20 Å**	
**Resolution**	**I/sigma**	**Resolution**	**I/sigma**
Inf - 3.00	7.76	Inf - 3.23	7.77
3.00 - 2.37	7.53	3.23 - 2.58	7.53
2.37 - 2.06	7.48	2.58 - 2.26	7.48
2.06 - 1.88	7.19	2.26 - 2.04	7.20
1.88 - 1.74	7.11	2.04 - 1.89	7.11
1.74 - 1.64	6.65	1.89 - 1.77	6.63
1.64 - 1.56	6.02	1.77 - 1.69	5.99
1.56 - 1.49	5.91	1.69 - 1.61	6.01
1.49 - 1.43	6.46	1.61 - 1.54	6.48
1.43 - 1.38	5.40	1.54 - 1.49	5.46
1.38 - 1.33	5.14	1.49 - 1.44	5.10
1.33 - 1.29	4.80	1.44 - 1.40	4.84
1.29 - 1.25	4.53	1.40 - 1.36	4.42
1.25 - 1.22	3.88	1.36 - 1.33	3.94
1.22 - 1.19	3.61	1.33 - 1.30	3.56

1.19 - 1.16	3.09	1.30 - 1.27	3.06
1.16 - 1.13	2.47	1.27 - 1.24	2.52
1.13 - 1.10	2.11	1.24 - 1.20	2.12
**Overall**	5.28	**Overall**	5.30

Table S6. Comparison of data statistics of SU-68 before and after resolution cutoff and simulation.

**0.60 Å (original)**		**0.70 Å**		**0.80 Å**		**0.90 Å**	
**Resolution**	**I/sigma**	**Resolution**	**I/sigma**	**Resolution**	**I/sigma**	**Resolution**	**I/sigma**
Inf - 1.63	8.67	Inf - 1.91	8.68	Inf - 2.14	8.65	Inf - 2.48	8.66
1.63 - 1.30	8.95	1.91 - 1.51	8.91	2.14 - 1.71	8.88	2.48 - 1.94	8.95
1.30 - 1.13	8.12	1.51 - 1.32	8.17	1.71 - 1.50	8.16	1.94 - 1.68	8.01
1.13 - 1.03	7.67	1.32 - 1.20	7.57	1.50 - 1.36	7.63	1.68 - 1.53	7.74
1.03 - 0.95	6.97	1.20 - 1.11	7.11	1.36 - 1.25	7.13	1.53 - 1.41	6.96
0.95 - 0.89	7.36	1.11 - 1.04	7.34	1.25 - 1.19	7.37	1.41 - 1.33	7.54
0.89 - 0.84	7.05	1.04 - 0.98	7.02	1.19 - 1.12	6.97	1.33 - 1.25	6.97
0.84 - 0.80	6.05	0.98 - 0.94	5.90	1.12 - 1.07	6.19	1.25 - 1.20	6.15
0.80 - 0.77	6.28	0.94 - 0.90	6.26	1.07 - 1.03	6.25	1.20 - 1.15	6.19
0.77 - 0.74	5.79	0.90 - 0.86	5.78	1.03 - 0.99	5.81	1.15 - 1.11	5.85
0.74 - 0.71	5.39	0.86 - 0.83	5.30	0.99 - 0.95	5.06	1.11 - 1.07	5.24
0.71 - 0.69	4.51	0.83 - 0.80	4.51	0.95 - 0.92	4.46	1.07 - 1.04	4.53
0.69 - 0.67	4.65	0.80 - 0.78	4.51	0.92 - 0.89	4.66	1.04 - 1.01	4.62
0.67 - 0.65	4.24	0.78 - 0.76	4.09	0.89 - 0.87	3.96	1.01 - 0.98	4.26
0.65 - 0.63	3.70	0.76 - 0.74	3.79	0.87 - 0.85	3.83	0.98 - 0.95	3.62
0.63 - 0.61	3.78	0.74 - 0.72	3.89	0.85 - 0.83	3.85	0.95 - 0.93	3.68
0.61 - 0.60	3.69	0.72 - 0.70	3.70	0.83 - 0.80	3.72	0.93 - 0.90	3.71
**Overall**	5.94	**Overall**	5.89	**Overall**	5.88	**Overall**	5.87

**1.00 Å**		**1.10 Å**		**1.20 Å**	
**Resolution**	**I/sigma**	**Resolution**	**I/sigma**	**Resolution**	**I/sigma**
Inf - 2.72	8.66	Inf - 2.84	8.58	Inf - 3.27	8.63
2.72 - 2.13	8.81	2.84 - 2.40	8.99	3.27 - 2.61	9.01
2.13 - 1.88	8.18	2.40 - 2.06	8.09	2.61 - 2.24	7.98
1.88 - 1.69	7.55	2.06 - 1.88	7.73	2.24 - 2.04	7.82
1.69 - 1.59	7.14	1.88 - 1.72	6.93	2.04 - 1.88	7.10
1.59 - 1.48	7.36	1.72 - 1.63	7.35	1.88 - 1.74	7.24

1.48 - 1.40	6.89	1.63 - 1.55	7.12	1.74 - 1.67	6.80
1.40 - 1.34	6.22	1.55 - 1.47	6.14	1.67 - 1.61	6.27
1.34 - 1.28	6.43	1.47 - 1.41	6.15	1.61 - 1.54	6.36
1.28 - 1.23	5.65	1.41 - 1.36	5.71	1.54 - 1.48	5.75
1.23 - 1.20	4.88	1.36 - 1.32	5.36	1.48 - 1.43	5.21
1.20 - 1.16	4.66	1.32 - 1.27	4.81	1.43 - 1.40	4.65
1.16 - 1.13	4.64	1.27 - 1.23	4.45	1.40 - 1.36	4.63
1.13 - 1.09	4.21	1.23 - 1.20	3.95	1.36 - 1.33	4.72
1.09 - 1.07	3.78	1.20 - 1.17	3.81	1.33 - 1.30	4.04
1.07 - 1.04	3.80	1.17 - 1.14	3.76	1.30 - 1.26	3.87
1.05 - 1.00	3.79	1.14 - 1.10	3.81	1.26 - 1.20	4.27
**Overall**	5.85	**Overall**	5.87	**Overall**	5.99

Table S7. Hydrogen bonding and corresponding distance between MPMD and framework of SU-8.

			N···O (Å)
N1A	*H1A1*	*O24*	3.13(2)
	H1A2	*O15*	2.81(2)
N2A	*H2A1*	*O30*	2.84(2)
	H2A2	*O6*	2.89(2)
N1B	*H1B1*	*O13*	2.95(2)
	H1B2	*O23*	2.80(2)
N2B	*H2B1*	*O28*	2.94(3)
	H2B1	*O5*	3.09(3)

Table S8. Hydrogen bonding and corresponding distance between TAEA and framework of SU-68.

			N···O (Å)
N1	*H1A*	*O5*	2.65(2)
	H1A	*O1*	3.13(2)
	H1B	*O6*	2.82(2)
N2	*H2A*	*O5*	2.70(2)
	H2A	*O2*	3.08(2)
	H2B	*O4*	2.88(2)
N3	*H3A*	*O5*	2.69(2)
	H3A	*O7*	3.09(2)
	H3B	*O3*	2.86(2)

References

(1) Wan, W.; Sun, J.; Su, J.; Hovmöller, S.; Zou, X. Three-Dimensional Rotation Electron Diffraction: Software *RED* for Automated Data Collection and Data Processing. *J. Appl. Crystallogr.* **2013**, *46* (6), 1863–1873.

(2) Kabsch, W. *XDS*. *Acta Crystallogr. D Biol. Crystallogr.* **2010**, *66* (2), 125–132.

(3) Ge, M.; Yang, T.; Wang, Y.; Carraro, F.; Liang, W.; Doonan, C.; Falcaro, P.; Zheng, H.; Zou, X.; Huang, Z. On the Completeness of Three-Dimensional Electron Diffraction Data for Structural Analysis of Metal–Organic Frameworks. *Faraday Discuss.* **2021**, *231* (0), 66–80.
(4) Sheldrick, G. M. A Short History of *SHELX*. *Acta Crystallogr. A* **2008**, *64* (1), 112–122.
(5) Huang, Z.; Ge, M.; Carraro, F.; Doonan, C.; Falcaro, P.; Zou, X. Can 3D Electron Diffraction Provide Accurate Atomic Structures of Metal–Organic Frameworks? *Faraday Discuss.* **2021**, *225* (0), 118–132.
(6) Grosse-Kunstleve, R. W.; Sauter, N. K.; Moriarty, N. W.; Adams, P. D. The Computational Crystallography Toolbox: Crystallographic Algorithms in a Reusable Software Framework. *J. Appl. Crystallogr.* **2002**, *35* (1), 126–136.
(7) Christensen, K. E.; Shi, L.; Conradsson, T.; Ren, T.; Dadachov, M. S.; Zou, X. Design of Open-Framework Germanates by Combining Different Building Units. *J. Am. Chem. Soc.* **2006**, *128* (44), 14238–14239.
